# Identification of Gut Microbiome Signatures Associated with the Kynurenine Pathway in Tryptophan Metabolism in Patients Undergoing Hemodialysis

**DOI:** 10.3390/microorganisms14102211

**Published:** 2026-10-01

**Authors:** Jih-Kai Huang, Ping-Hsun Wu, Zhao-Feng Chen, Po-Yu Liu, Yun-Shiuan Chuang, Mei-Chuan Kuo, Yi-Wen Chiu, Ping-Chi Hsu, Tien-Hsiang Kuo, Yi-Ting Lin

**Affiliations:** 1 Department of Emergency Medicine, Kaohsiung Medical University Hospital, Kaohsiung Medical University, Kaohsiung 807, Taiwan; eric86425@yahoo.com.tw; 2 Division of Nephrology, Department of Internal Medicine, Kaohsiung Medical University Hospital, Kaohsiung Medical University, Kaohsiung 807, Taiwan; 970392@kmuh.org.tw (P.-H.W.); chiuyiwen@kmu.edu.tw (Y.-W.C.); 3 Center for Big Data Research, Kaohsiung Medical University, Kaohsiung 807, Taiwan; kinkipag@gmail.com; 4 Research Center for Precision Environmental Medicine, Kaohsiung Medical University, Kaohsiung 807, Taiwan; pchsu@nkfust.edu.tw; 5 Faculty of Medicine, College of Medicine, Kaohsiung Medical University, Kaohsiung 807, Taiwan; 6 Biomedical Artificial Intelligence Academy, Kaohsiung Medical University, Kaohsiung 807, Taiwan; 7 Department of Horticulture and Landscape Architecture, National Taiwan University, Taipei 106319, Taiwan; ivan.chen1966@gmail.com; 8 School of Medicine, College of Medicine, National Sun Yat-sen University, Kaohsiung 804201, Taiwan; poyu.liu@gmail.com; 9 Department of Family Medicine, Kaohsiung Medical University Hospital, Kaohsiung Medical University, Kaohsiung 807, Taiwan; 10 Department of Safety, Health and Environmental Engineering, National Kaohsiung University of Science and Technology, Kaohsiung 807, Taiwan; 11 Department of Public Health, College of Health Sciences, Kaohsiung Medical University, Kaohsiung 807, Taiwan; 12 School of Medicine, Kaohsiung Medical University, Kaohsiung 807, Taiwan; thomas97747@gmail.com; 13 Division of Family Medicine and Primary Care, Department of Neurobiology, Care Sciences and Society, Karolinska Institute, 14183 Stockholm, Sweden; 14 Big Data Center, China Medical University Hospital, China Medical University, Taichung 404, Taiwan

**Keywords:** chronic kidney disease, end-stage renal disease, gut microbiota, hemodialysis, tryptophan metabolism

## Abstract

Patients with chronic kidney disease (CKD) and hemodialysis (HD) often experience gut microbiota dysbiosis, which may influence tryptophan metabolism through the kynurenine pathway. We aimed to investigate the specific gut microbiota signatures associated with metabolites involved in the kynurenine pathway in HD patients. In this cross-sectional study, we analyzed serum metabolite concentrations and fecal shotgun metagenomic profiles from 85 patients receiving maintenance hemodialysis at Kaohsiung Medical University Hospital. Fecal samples were analyzed using shotgun metagenomic sequencing to characterize microbiome composition and functional pathways. We measured kynurenine pathway metabolites (kynurenine, kynurenic acid, nicotinuric acid, and trigonelline) by liquid chromatography tandem mass spectrometry and analyzed their associations with microbial species abundance and genomic metabolic modules (GMM). Several bacterial species displayed associations with kynurenine pathway metabolites. *Ruminococcus gnavus* and *Eubacterium* sp. CAG:180 demonstrated correlations with kynurenine and kynurenic acid levels, respectively; however, these associations did not persist after adjustment. Distinct levels of metabolites corresponded to distinct patterns of genomic metabolic modules. High kynurenine levels correlated with increased pathways for pectin and arginine degradation. Elevated nicotinuric acid levels were associated with enhanced pathways for anaerobic fatty acid beta-oxidation and propionate production. Trigonelline levels correlated with homocysteine metabolism and various amino acid degradation pathways. Specific microbial taxa and predicted functional modules were associated with circulating kynurenine- and niacin-related metabolites. These cross-sectional findings are hypothesis-generating and do not establish directionality, causality, or therapeutic efficacy. Interpretation is limited by the modest sample size and the absence of a non-hemodialysis control group.

## 1. Introduction

Chronic kidney disease (CKD) is a major public health issue in Taiwan, with high prevalence and significant socioeconomic impact [1,2]. CKD or hemodialysis (HD) are both associated with numerous comorbidities, including cardiovascular complications, systemic inflammation, and metabolic disturbances, all of which contribute to increased morbidity and mortality among these patients [3,4]. The high burden of comorbidities underscores the complex challenges in managing patients receiving maintenance hemodialysis and highlights the need to explore underlying mechanisms that may exacerbate these adverse health outcomes [3,4].

One emerging area of interest in HD patient health is the role of gut microbiota dysbiosis [5]. Dysbiosis, characterized by significant alterations in microbial composition and diversity, is commonly observed in individuals receiving maintenance hemodialysis and is believed to contribute to the inflammation and metabolic dysfunction frequently seen in this population [6]. Changes in gut microbiota may affect various metabolic pathways within the host, particularly those involving tryptophan metabolism [7]. The kynurenine pathway, which is the primary route for tryptophan catabolism, plays an essential role in immune regulation, neuroprotection, and cellular energy production [8]. Dysregulation of this pathway, observed in numerous chronic conditions, could further complicate health outcomes for patients receiving maintenance hemodialysis by contributing to both inflammation and immune dysregulation [9]. We selected kynurenine because it is the central intermediate of the kynurenine pathway and kynurenic acid because it is a major downstream branch product. Nicotinuric acid and trigonelline were included as niacin-related metabolites that may capture adjacent aspects of niacin handling, dietary exposure, and host–microbial metabolism. Together, these analytes provide complementary information across the kynurenine–niacin metabolic axis; nicotinuric acid and trigonelline are not interpreted as direct canonical intermediates of the kynurenine pathway.

Although dysregulation of tryptophan metabolism and gut microbial composition are both common in hemodialysis, it remains unclear whether circulating kynurenine- and niacin-related metabolites are associated with species-level microbial composition and predicted functional capacity in this population. We hypothesized that metabolite concentrations would be associated with a limited set of microbial species and genomic metabolic modules even in the absence of broad differences in alpha or beta diversity. We therefore integrated targeted serum metabolite measurements with fecal shotgun metagenomics to characterize these associations in patients receiving maintenance hemodialysis.

## 2. Materials and Methods

Detailed protocols for participant enrollment, LC–MS/MS metabolite quantification, metagenomics sequencing/bioinformatics, and statistical modeling are provided in Supplementary Methods (S1–S6).

### 2.1. Study Population

We enrolled 85 patients receiving maintenance hemodialysis from Kaohsiung Medical University Hospital, Taiwan (August 2017–February 2018). Patients with active malignancy or antibiotic use within 3 months were excluded. This exploratory analysis included all participants for whom paired serum metabolite measurements and fecal metagenomic profiles were available. The analytic sample size was therefore determined by the available cohort and paired biospecimens; no a priori microbiome-specific power calculation was performed. All participants received maintenance hemodialysis using high-flux dialyzers for 3.5–4 h per session, three times weekly. The study was approved by the Institutional Review Board of Kaohsiung Medical University (KMUHIRB-E(I)-20160095 and KMUHIRB-E(I)-20180118), and written informed consent was obtained from all participants (details in Supplementary Methods S1).

### 2.2. Comorbidities and Laboratory and Clinical Variables

Demographics, comorbidities, medication use (including PPI), and laboratory variables were extracted from medical records. Diabetes mellitus, hypertension, and coronary artery disease were defined using prespecified clinical criteria. Fasting blood samples were collected before scheduled HD sessions; albumin and hs-CRP were obtained from results recorded within 30 days prior to HD (definitions and data collection details in Supplementary Methods S1).

### 2.3. Kynurenine Metabolites Measurement

Serum kynurenine-pathway metabolites (kynurenine, kynurenic acid, nicotinuric acid, and trigonelline) were quantified using targeted liquid chromatography–tandem mass spectrometry (LC–MS/MS). Sample preparation, calibration/quality control procedures, and instrument settings are described in Supplementary Methods S2.

### 2.4. Microbiome Analysis

Stool specimens were frozen immediately after collection and transported in insulated containers to Germark Biotechnology (Taichung, Taiwan) within 24 h. Microbial DNA was isolated with the QIAamp Fast DNA Stool Mini Kit (QIAGEN GmbH, Hilden, Germany). DNA quantity and purity were assessed by NanoDrop ND-1000 spectrophotometry (Thermo Fisher Scientific/NanoDrop, Wilmington, DE, USA), and integrity was evaluated by agarose gel electrophoresis before storage at −80 °C. Approximately 500 ng of DNA per sample was fragmented to a target length of about 350 bp with a Covaris S2 instrument (Covaris, Inc., Woburn, MA, USA). Libraries were prepared with the Illumina DNA Prep kit (Illumina, Inc., San Diego, CA, USA) and sequenced as 150-bp paired-end reads on an Illumina NovaSeq 6000 platform (Illumina, Inc., San Diego, CA, USA). Sequencing depth achieved an average throughput of 8–9 Gb per sample (ranging from 40 to 85 million raw reads per sample). Raw paired-end reads were processed with KneadData v0.7.433. Adapter removal and quality trimming were performed with Trimmomatic v0.39. Bowtie2 v2.4.2 alignments to the human hg38 and PhiX references were used to remove host and control reads. Low- complexity and repetitive reads were removed with Komplexity v0.3.6 using default settings. Quality-controlled reads were assembled with MEGAHIT v1.0. Read-level taxonomic profiling used Sourmash v2.0, and contig-level taxonomic assignment used the MMseqs2 easy-taxonomy workflow. Retained reads were aligned back to contigs with Bowtie2 v2.4.2. Open reading frames were predicted and annotated with Prokka v1.14.5, including COG and Enzyme Commission annotations. KEGG Orthology assignments were generated with MMseqs2 easy-search, and pathway presence was reconstructed with MinPath v1.1 using KEGG release 112.1 and MetaCyc/Pathway Tools release 28.5 definitions. Metagenome-assembled genomes were recovered with MetaBAT v2.17 and assessed with CheckM v1.1.6. High-quality genomes were defined as ≥90% complete with ≤5% contamination; medium-quality genomes were defined as ≥50% complete with ≤10% contamination, and genomes not meeting these thresholds were excluded. For species-level analyses, counts were converted to relative abundances. Features with a mean relative abundance across the 85 samples of 1 × 10^−5^ or less were removed. Non-bacterial records and records lacking complete taxonomic annotation were also excluded, after which retained features were aggregated at the species level. This procedure yielded 867 species for feature-wise analyses. Analyses were performed in R v4.2.2.

### 2.5. Statistical Analysis

Analyses were conducted in R. Microbiome profiles were transformed to relative abundance and filtered to remove rare taxa, then aggregated at the species level (Supplementary Methods S5). For each metabolite, participants were categorized into high vs. low groups by the median concentration. Baseline characteristics were compared using t-tests or Wilcoxon rank-sum tests for continuous variables and chi-square tests for categorical variables. Alpha diversity (Shannon, Simpson, inverse Simpson) and beta diversity (Bray–Curtis; PCoA) were calculated at the species level; group differences were evaluated using unadjusted PERMANOVA with 999 free permutations. For each metabolite, age- and sex-adjusted partial Spearman correlations were calculated across the 867 retained species and, separately, across 100 genomic metabolic modules. Within each metabolite-by -data-layer family, *p*-values were adjusted using the Benjamini–Hochberg procedure. Associations with q < 0.05 were considered FDR-significant. Results with 0.05 ≤ q < 0.20 are reported only as exploratory. LEfSe (version 1.1.2) analyses used an LDA score threshold of 2.0 as an exploratory feature-ranking procedure; because default LEfSe *p*-values do not control the false discovery rate, LEfSe findings are reported with nominal *p*-values and LDA scores and are not described as FDR-significant. After correction, no species-level association met q < 0.05 for any of the four metabolites. Among the GMM analyses, lactate consumption II was positively associated with nicotinuric acid after age and sex adjustment (partial Spearman ρ = 0.384, *p* = 3.66 × 10^−4^, q = 0.0366; *n* = 85). Propionate production II was exploratory (ρ = 0.329, *p* = 0.00254, q = 0.127); no other metabolite-specific GMM family contained an association with q < 0.05 For the historical screening analysis, LEfSe was applied using log10p transformation, Wilcoxon and Kruskal–Wallis cutoffs of 0.05, multigroup stratification, and an LDA-score cutoff of 2.1. Among the LEfSe markers, taxa with a nominal equal-variance two-sample comparison *p* < 0.05 were carried forward. To calculate the reported descriptive percentages, the retained species were entered jointly into a separate ordinary least-squares model in all 85 participants. We report the conventional full-sample coefficient of determination (R^2^ = 1 − SSE/SST). Because both screening and final estimation were data-derived within this cohort, these values describe apparent in-sample fit and should not be interpreted as out-of-sample predictive performance or causal variance attributable to the microbiome.

## 3. Results

### 3.1. Baseline Characteristics

The baseline characteristics are demonstrated for four metabolites of the kynurenine pathway in Table 1. The mean age, gender, presence of DM, HTN, PPI use, Kt/V (D), hs-CRP, and hemodialysis vintage were similar between the high and low concentration groups for the four metabolites. Presence of CAD was significantly different between the high and low groups for kynurenine (*p* = 0.02), and albumin level revealed a significant difference for kynurenic acid (*p* = 0.037), as shown in Table 1.

### 3.2. Differential Abundance and Diversity of Gut Microbiota in Response to Kynurenine Metabolite Levels

**Figure 1:**
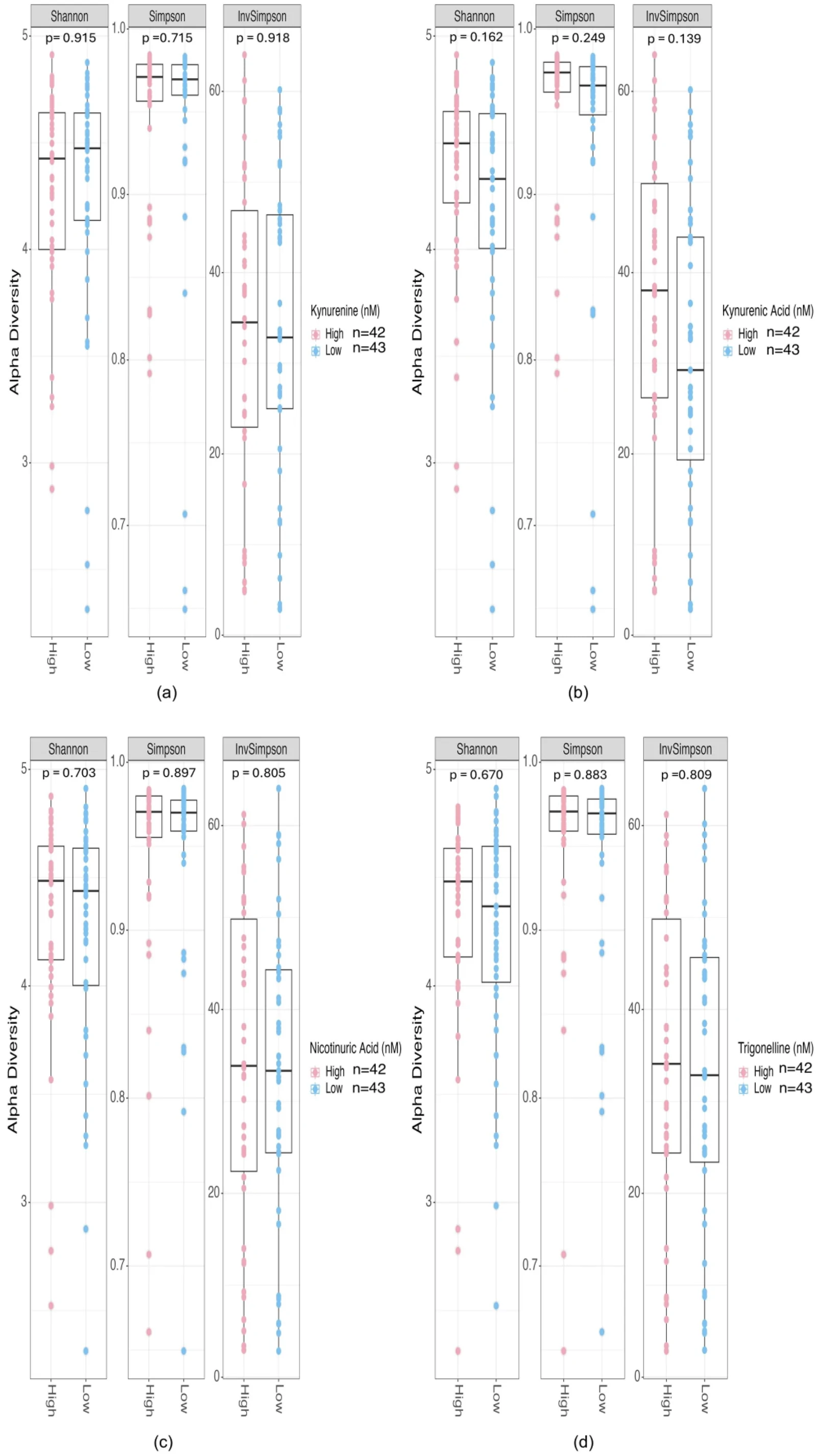
Alpha diversity corresponding to different levels of kynurenine metabolites from the tryptophan pathway: (**a**) kynurenine; (**b**) kynurenic acid; (**c**) nicotinuric acid; (**d**) trigonelline.

We demonstrated the differential abundance of microbiota genera and species based on the concentrations of kynurenine pathway metabolites. Each figure showed the microbial distribution associated with distinct metabolites, specifically kynurenine (Supplementary Figure S1), kynurenic acid (Supplementary Figure S2), nicotinuric acid (Supplementary Figure S3), and trigonelline (Supplementary Figure S4). The computation of the alpha diversity indices, as shown in Figure 1, which indicate the microbial evenness and richness within each patient group, showed that no significant difference was found between high and low levels of all four metabolites, based on Shannon, Simpson, and InvSimpson indices.

**Figure 2:**
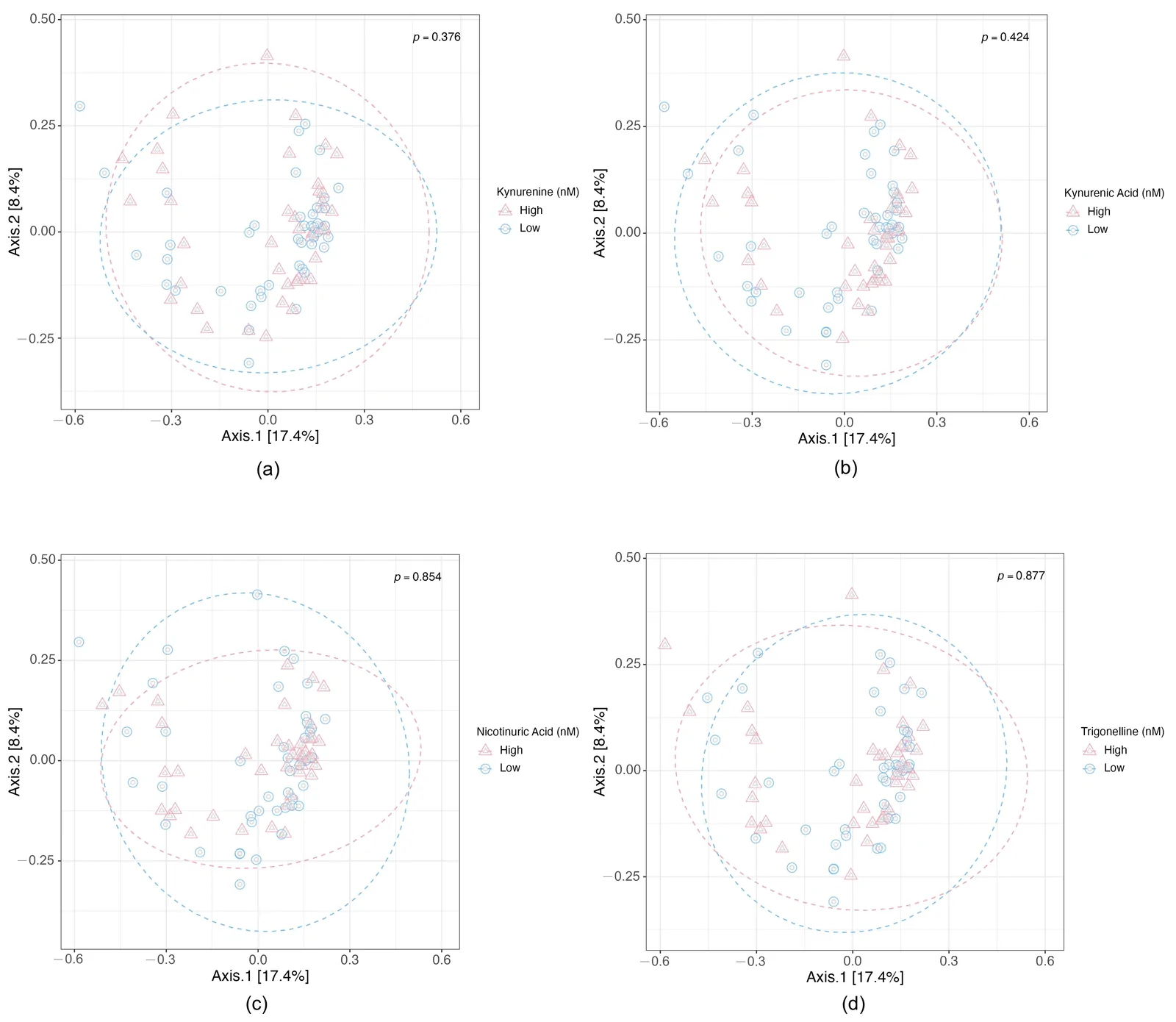
Beta diversity corresponding to different levels of kynurenine metabolites from the tryptophan pathway: (**a**) kynurenine; (**b**) kynurenic acid; (**c**) nicotinuric acid; (**d**) trigonelline.

PCoA of beta diversity, which identifies inter-group dissimilarities, showed no significant difference in composition associated with varying metabolite levels (Figure 2). Principal coordinate analysis was based on species-level Bray–Curtis dissimilarity. Axis 1 and Axis 2 explained 17.4% and 8.4% of the ordination variance, respectively. Unadjusted PERMANOVA with 999 free permutations showed no evidence of separation between median-defined groups: kynurenine, R^2^ = 0.0121, F = 1.015, *p* = 0.376; kynurenic acid, R^2^ = 0.0118, F = 0.994, *p* = 0.424; nicotinuric acid, R^2^ = 0.0086, F = 0.724, *p* = 0.854; and trigonelline, R^2^ = 0.0086, F = 0.718, *p* = 0.877. These models did not adjust for clinical covariates. The community structures were similar across different kynurenine (*p* = 0.376), kynurenic acid (*p* = 0.424), nicotinuric acid (*p* = 0.854), and trigonelline (*p* = 0.877) levels, indicating that the concentrations of those kynurenine metabolites were not significantly associated with the microbiota profiles. PERMANOVA did not identify global species-level community separation between the high and low metabolite groups. This omnibus test evaluates the full Bray–Curtis distance matrix and may have limited sensitivity to sparse changes confined to a small subset of taxa. The subsequent species- and module-level analyses therefore address a different, feature-specific hypothesis and are not intrinsically inconsistent with the absence of global beta-diversity separation. Because many features were tested and no species-level partial correlation met q < 0.05, these localized signals are interpreted as exploratory.

### 3.3. Association Between Kynurenine Concentration, Gut Microbiota Composition, and Metabolic Pathway

The association between kynurenine and gut microbiota composition is shown in Supplementary Figure S5 by LEfSe. Further comparison of the standardized relative abundance between high and low concentrations of kynurenine is shown in Supplementary Figure S6. After Benjamini–Hochberg correction across 867 species within each metabolite, no species-level partial correlation met q < 0.05. Eubacterium sp. CAG:180 showed an exploratory inverse association with kynurenine after adjustment for age and sex (partial Spearman ρ = −0.412, *p* = 1.19 × 10^−4^, q = 0.103; n = 85). The full-sample selected-species linear model for kynurenine yielded a full-sample R^2^ of 0.318 (31.8%). GMM analysis showed distinct functional profiles between high and low kynurenine groups, with enrichment of carbohydrate- and amino-acid–related modules in the high group (Supplementary Figure S7).

### 3.4. Influence of Gut Microbiota and Metabolic Pathway on Kynurenic Acid Concentration

The relationship between kynurenic acid and microbiota composition was demonstrated in Supplementary Figure S8 by LEfSe. Further comparison of the standardized association between high and low concentrations of kynurenic acid is shown in Supplementary Figure S9. The selected-species linear model for kynurenic acid had a full-sample R^2^ of 0.202 (20.2%). GMM profiles differed modestly by kynurenic acid status; selected amino-acid degradation modules were enriched in the low group (Supplementary Figure S10).

### 3.5. Association Between Nicotinuric Acid Concentration, Gut Microbiota Composition, and Metabolic Pathway

The association between nicotinuric acid and gut microbiota composition was revealed in Supplementary Figure S11 by LEfSe. Further comparison of standardized relative abundance between high and low nicotinuric acid levels is shown in Supplementary Figure S12. In the 100-module GMM family, lactate consumption II was positively associated with nicotinuric acid (ρ = 0.384, *p* = 3.66 × 10^−4^, q = 0.0366; n = 85). The selected-species linear model for nicotinuric acid had a full-sample R^2^ of 0.016 (1.6%). GMM analysis showed distinct functional differences by nicotinuric acid level, including modules related to fatty-acid metabolism and short-chain fatty-acid production (Supplementary Figure S13).

### 3.6. Interaction Between Trigonelline Concentration, Gut Microbiota Composition, and Metabolic Pathway

The interplay between trigonelline and gut microbiota composition is shown in Supplementary Figure S14 by LEfSe. A further comparison of the standardized abundance between high and low concentrations of trigonelline was revealed in Supplementary Figure S15. The selected-species linear model for trigonelline had a full-sample R^2^ of 0.058 (5.8%). Trigonelline groups showed differences in multiple metabolic modules, including sulfur amino acid and energy-related pathways (Supplementary Figure S16).

### 3.7. Sensitivity Analysis

Sensitivity analyses repeated the feature-wise partial Spearman analyses after adjustment for age, sex, diabetes, hypertension, coronary artery disease, proton-pump-inhibitor use, and serum albumin. Benjamini–Hochberg correction was repeated separately within each metabolite and data layer. These expanded models are interpreted as sensitivity analyses rather than independent confirmatory tests. For Eubacterium sp. CAG:180, the inverse association with kynurenine attenuated from ρ = −0.412 (*p* = 1.19 × 10^−4^, q = 0.103) in the age- and sex-adjusted model to ρ = −0.317 (*p* = 0.00492, q = 0.533) after expanded adjustment. Frisingicoccus caecimuris had the smallest expanded-model q-value for kynurenine (ρ = −0.415, *p* = 1.73 × 10^−4^, q = 0.150), but this also remained exploratory. Thus, no species association remained FDR-significant after adjustment for the broader clinical covariate set.

## 4. Discussion

In the present study, we demonstrated the gut microbiota profile and the functional pathways in ESRD patients under regular HD by shotgun metagenomic sequencing. We analyzed the variations in the gut microbiota and metabolic modifications correlating with the levels of kynurenine and its metabolites. To our knowledge, it is the first study to reveal the association between kynurenine metabolism and its derivatives’ biosynthesis, functional module, and gut microbiota in ESRD patients under regular HD. Our findings demonstrate cross-sectional associations rather than a directional host–microbe mechanism. The identified taxa may contribute to metabolite variation, may respond to the same host environment, or may mark unmeasured dietary, pharmacologic, or clinical factors. The functional modules were inferred from genomic content and indicate metabolic potential, not pathway expression, flux, or metabolite production in vivo.

### 4.1. Microbiota Composition

In the present study, several species, including *Oscillospiraceae bacterium*, *Ruminococcus gnavus*, *Ruthenibacterium lactatiformans*, *Ruminococcus torques*, *Frisingicoccus caecimuris*, *Clostridioides difficile*, and *Eubacterium* sp. CAG: 180, were associated with lower levels of kynurenine. Among these bacteria, *Ruminococcus gnavus* is an anaerobic, Gram-positive bacterium. Interestingly, there is a positive correlation between kynurenine and the degradation of rhamnose, some amino acids, and glucose. This may prove the interaction between *Ruminococcus gnavus* and functional enzyme modification through kynurenine. In the gut microbiome, *Eubacterium* sp. CAG:180 is associated with inflammatory protein levels [10]. On the other hand, kynurenine was proven to act as an anti-inflammatory molecule by the induction from pro-inflammatory cytokines in several studies [11,12]. The down-regulation of kynurenine from those bacteria may be a crucial mechanism to promote the inflammatory process. Moreover, previous studies showed that *Oscillospiraceae bacteria* and *Ruminococcus torques* down-regulate the level of tryptophan, the origin of kynurenine [13]. This outcome may explain the negative association of kynurenine with these two bacteria in the present study. Since kynurenine is regarded as a uremic toxin, its level increased significantly in ESRD patients and could have altered concentration under regular HD [14]. The interplay between kynurenine and its functional modification by the effects of microbiota is important in this group because gut microbiota play a significant role in kynurenine synthesis [15,16]. Our study offered a novel aspect for varying abundance in species to show the functional effects in ESRD patients based on the level of kynurenine levels. However, the specific mechanism remains unknown and controversial.

For kynurenic acid, *Bacteroides ovatus* and *Bacteroides caccae* CAG:21 are associated with higher levels of kynurenic acid. *Alistipes communis* and *Eubacterium* sp. CAG:180 are linked to lower levels of kynurenic acid. The pro-inflammatory characteristics of *Eubacterium* sp. CAG:180 have been discussed previously. On the contrary, kynurenic acid was shown to regulate mucosal protective effects in the gut [17]. Thus, *Eubacterium* sp. CAG:180 may be related to the inflammatory process in the gut. Kynurenic acid also showed protective effects against inflammatory bowel diseases (IBD), such as ulcerative colitis [18]. Previous studies have revealed an enrichment of *Bacteroides* after treatment with kynurenic acid, and the severity of IBD was correlated with decreasing diversity of *Bacteroides* sp. [18,19]. Experimental studies in colitis models provide biological context for several taxa and pathways discussed here, but they address a different disease setting and cannot establish the function of these organisms in patients receiving hemodialysis. In the present cohort, we observed associations only; no intestinal inflammatory phenotype or microbial intervention was evaluated. The up- and down-regulation of kynurenic acid with *Bacteroides* spp., *Alistipes communis*, and *Eubacterium* sp. CAG:180 showed possible mechanisms of functional modulation in those ESRD patients.

Regarding the concentration of nicotinuric acid, *Bacteroides* sp. CAG:754, *Bacteroides* sp. 214, and *Ruminococcus torques* correlate with higher levels. Nicotinuric acid is a glycine conjugate of nicotinic acid and may reflect aspects of niacin handling. Although the kynurenine pathway can contribute to de novo NAD biosynthesis, this study did not measure NAD^+^/NADP^+^ pools, redox state, oxidative damage markers, enzyme activity, or pathway flux. The observed associations between nicotinuric acid and microbial features therefore should not be interpreted as evidence of a microbiome-mediated NAD^+^ or oxidative -stress mechanism.

The combination of preserved global community structure and localized feature-level associations suggests that variation in these metabolites may relate to a limited set of microbial components rather than wholesale community reconfiguration. Such a pattern could be biologically relevant if particular organisms or functions disproportionately participate in tryptophan- or niacin-related metabolism. However, directionality is unknown: the microbial features could contribute to metabolite variation, respond to the host metabolic environment, or share underlying causes. These findings therefore do not establish that modifying the identified features would alter clinical outcomes.

This study has several limitations. Its cross-sectional design precludes temporal or causal inference and is susceptible to reverse causation. The modest sample size limits precision and power, particularly after correction for multiple testing, and the absence of a non-hemodialysis control group prevents us from determining whether the observed patterns are specific to hemodialysis. Dietary intake, including tryptophan, coffee and other sources of trigonelline, pectin, and fermentable substrates that influence short-chain-fatty-acid production, was not collected, leaving important potential confounding. Residual confounding by medications, comorbidities, nutritional status, and dialysis-related factors is also possible. Predicted genomic modules reflect functional potential rather than transcription, metabolite flux, or host exposure, and no experimental validation was performed. Finally, the absence of a global PERMANOVA difference suggests that the measured metabolites are not dominant determinants of overall community configuration, although smaller feature-specific associations may still be present.

Trigonelline up-regulation is associated with the bacteria *Oscillospiraceae*, while down-regulation was related to *Parabacteroides johnsonii*. Trigonelline is abundant in coffee beans and has antimicrobial, anti-lipidemic, and antioxidant effects [20]. Trigonelline is also produced by gut microbiota from the metabolism of tryptophan and represents the metabolism of choline [21]. It has several anti-inflammatory effects that reduce renal fibrosis, preventing hyperlipidemia and hyperglycemia based on the homeostasis of gut microbiota [21,22,23]. A previous study showed an increase in *Oscillospiraceae* in mice with high-fat or high-sugar diets [24]. However, the detailed mechanism and further effects remained unknown. Our study provided a possible interplay between trigonelline and *Oscillospiraceae*. On the other hand, *Parabacteroides johnsonii* could catalyze the conversion of polymers to simple sugars and is enriched in patients with alopecia [25]. However, the effect of trigonelline on this pathway remains unknown.

### 4.2. Variations in Functional Pathways of Gut Microbiota

In this study, we showed the altered metabolic function of gut microbiota in ESRD patients under regular HD, followed by the different levels of kynurenine and its derivatives. To our knowledge, it is the first study to reveal the functional variations based on kynurenine metabolites in such groups. Some studies have shown functional modifications in gut microbiota depending on kynurenine in animals, and different microbiota effects on kynurenine metabolism in CKD patients [11]. Nonetheless, the sample size and the number of studies were still limited. The present study provided the latest development in this issue.

Increasing kynurenine concentration was related to pectin degradation I and II, lactate consumption I, pentose phosphate pathway, glutamate degradation II, lactaldehyde degradation, aspartate degradation I and IV, arginine degradation I, and rhamnose degradation. On the contrary, low kynurenine level was associated with triacylglycerol degradation, glutamate degradation III, and pyruvate dehydrogenase complex. Dietary pectin supplements, especially high methyl-esterified pectin, showed a strong ability to improve gut health in animal models. However, low methyl-esterified pectin had detrimental effects due to interference with gut microbiota and kynurenine metabolism [26]. Our study showed the possible mechanism of functional modification between kynurenine and pectin. Arginine can be metabolized by arginase [1], which acts as an immune regulator and sometimes activates with IDO-1 [27]. We also found a positive correlation between kynurenine levels and arginine degradation. This outcome may support the cooperation between arginase 1 and IDO-1 in immunoregulation. The present study showed increased lactate consumption in elevated kynurenine, which may offer evidence of the interaction between these two molecules. Gut microbiota may play an important role in this mechanism based on our findings. The kynurenine/ tryptophan ratio was negatively correlated with fatty acid oxidation in the past animal study [28]. Another animal study showed the neuroprotective effect of kynurenine via decreasing L-glutamate levels [29]. However, we found that glutamate degradation may be linked to both increasing and decreasing kynurenine levels. Further study may be needed to develop the detailed association between glutamate and kynurenine.

The decreasing kynurenic acid concentrations showed enrichment in negatively charged amino acid degradation and glutamate degradation III. Negatively charged amino acids, including aspartate and glutamate, form several protein molecules such as the nicotinic receptor [30]. Kynurenic acid synthesis is one of the three major metabolic routes of kynurenine [31]. The increasing kynurenic acid formation may down-regulate the production of other metabolites, including NAD+ that involves the effect of nicotinic acid [31]. There may be some relationship between the kynurenic level and the NAD+ production via a module of aspartate and glutamate. Further study is needed for a more detailed evaluation. On the other hand, past studies also demonstrated that kynurenic acid contributed to the inhibition of glutamate release and receptor activity in neural transmission [32]. Our study may provide an additional mechanism in this relationship.

Increased nicotinuric acid level was associated with anaerobic fatty acid beta-oxidation, succinate conversion to propionate, lactate consumption II, propionate production II, lactose and galactose degradation. On the other hand, decreased nicotinuric acid concentration was related to tyrosine degradation I, glyoxylate bypass, pectin degradation I, pectine degradation II, acetate to acetyl-CoA, mannose degradation, butyrate production I, pentose phosphate pathway (non-oxidative branch), xylose degradation, arabinose degradation, arginine degradation IV, glycerol degradation II, and threonine degradation II. As mentioned above, the kynurenine pathway is the main source of NAD+ production [32]. Succinate and succinyl-CoA, the byproducts of the TCA cycle, can further be converted to propionate [33]. Therefore, the outcomes of our study provide the relationship between nicotinuric acid and the functions of NAD+. Interestingly, a previous study has found a positive correlation between propionate and the phylum *Bacteroidetes* [33]. The abundant *Bacteroides* sp. in the up-regulation of nicotinuric acid, shown in the present study, may offer detail association between them. NAD+ may also be phosphorylated to NADP+, and promote PPP [34]. Thus, decreased nicotinuric acid levels induced by NADP+ production can still cause the degradation of glucose and its upstream products. Furthermore, decreased NAD+ concentrations also result in dysfunction of redox homeostasis, which leads to protein oxidation and cell damage [35]. Our study demonstrated the possible interplay between the variant functional status of microbiota in declining nicotinuric acid and the impacts on the human body.

Varying trigonelline levels also modulate the functional status of bacteria. Higher trigonelline was correlated with cysteine biosynthesis, homocysteine degradation, glycerol degradation I, pyruvate dehydrogenase complex, fructan degradation, and lysine degradation II. On the contrary, lower trigonelline was related to glutamate degradation III, glutamine degradation I, glycine degradation, pentose phosphate pathway, and succinate production. Trigonelline is a byproduct in choline metabolism and is mainly formed during the conversion of S-adenosylmethionine to S-adenosylhomocysteine [21,36]. S-adenosylhomocysteine is further converted into homocysteine and may be catabolized to cysteine [37]. Hyperhomocysteinemia is regarded as a risk factor for endothelial damage [38]. The protective role of trigonelline in the cardiovascular system may be attributed to homocysteine degradation, which is revealed to be correlated with increasing trigonelline in our study. Moreover, trigonelline is produced by nicotinic acid and is further involved in the formation of glutathione, which features antioxidant characteristics [21,22]. Therefore, the variant biochemical function depending on the levels of trigonelline showed a similar pattern to nicotinuric acid.

## 5. Conclusions

In this cross-sectional study of 85 patients receiving maintenance hemodialysis, circulating kynurenine- and niacin-related metabolites were associated with selected microbial species and predicted functional modules, whereas alpha diversity and overall beta diversity did not differ between median-defined groups. Given the modest sample size, absence of non-hemodialysis controls, unavailable dietary data, and lack of experimental validation, these findings are exploratory and do not establish microbial effects on host metabolism. Larger longitudinal studies with dietary characterization, external validation, and mechanistic or interventional experiments are needed before the clinical or therapeutic relevance of these associations can be determined.

## Figures and Tables

**Table 1:** Demographic and clinical characteristics of study participants in the kynurenine pathway analysis with corresponding metabolite data (N = 85).

Parameters/Groups n (%) or Mean (SD) or Median [Q1; Q3]	Kynurenine (nM)		*p*	Kynurenic Acid (nM)		*p*	Nicotinuric Acid (nM)		*p*	Trigonelline (nM)		*p*
	High (N = 42)	Low (N = 43)		High (N = 42)	Low (N = 43)		High (N = 42)	Low (N = 43)		High (N = 42)	Low (N = 43)	
Age	59.9 (9.74)	61.1 (11.7)	0.619	58.3 (8.69)	62.7 (12.1)	0.057	60.1 (12.1)	60.9 (9.38)	0.738	58.7 (9.83)	62.3 (11.4)	0.131
Female	19 (45.2%)	17 (39.5%)	0.755	18 (42.9%)	18 (41.9%)	1.000	17 (40.5%)	19 (44.2%)	0.899	17 (40.5%)	19 (44.2%)	0.899
DM	10 (23.8%)	18 (41.9%)	0.124	10 (23.8%)	18 (41.9%)	0.124	13 (31.0%)	15 (34.9%)	0.877	13 (31.0%)	15 (34.9%)	0.877
HTN	30 (71.4%)	37 (86.0%)	0.166	31 (73.8%)	36 (83.7%)	0.394	31 (73.8%)	36 (83.7%)	0.394	32 (76.2%)	35 (81.4%)	0.748
CAD	4 (9.52%)	14 (32.6%)	0.020	7 (16.7%)	11 (25.6%)	0.459	11 (26.2%)	7 (16.3%)	0.394	10 (23.8%)	8 (18.6%)	0.748
PPI	8 (19.0%)	5 (11.6%)	0.516	6 (14.3%)	7 (16.3%)	1.000	7 (16.7%)	6 (14.0%)	0.963	6 (14.3%)	7 (16.3%)	1.000
Albumin	3.92 (0.31)	3.90 (0.39)	0.786	3.99 (0.33)	3.83 (0.36)	0.037	3.95 (0.32)	3.87 (0.37)	0.342	3.92 (0.31)	3.90 (0.39)	0.805
Kt/V (D)	1.54 (0.22)	1.53 (0.20)	0.949	1.30 (0.14)	1.31 (0.18)	0.702	1.53 (0.18)	1.54 (0.24)	0.855	1.55 (0.18)	1.53 (0.24)	0.678
HS-CRP	4.36 (5.18)	4.61 (6.49)	0.845	4.27 (4.64)	4.71 (6.91)	0.733	3.79 (4.51)	5.20 (6.93)	0.273	4.42 (5.12)	4.56 (6.54)	0.918
Hemodialysis Vintage	89.8 (67.0)	93.3 (77.8)	0.823	95.0 (74.2)	88.2 (71.1)	0.669	101 (69.6)	82.7 (74.5)	0.252	89.5 (69.9)	93.6 (75.3)	0.797
Metabolites (nM)	5752 [5080; 6609]	3865 [3193; 4317]	<0.001	3801 [3587; 4353]	2174 [1714; 2534]	<0.001	18.6 [8.86; 51.6]	4.08 [3.04; 5.07]	<0.001	404 [303; 579]	162 [114; 194]	<0.001

Note: SD: standard deviation; Q1: the first quartile; Q3: the third quartile; DM: diabetes mellitus; HTN: hypertension; CAD: coronary artery disease; PPI: proton pump inhibitor; HS-CRP: high-sensitivity C-reactive protein.

## Data Availability

Due to ethical restrictions imposed by the Institutional Review Board of Kaohsiung Medical University Hospital (IRB Nos. KMUHIRB-E(I) 20160095 and KMUH IRB-E(I)-20180118), the raw metagenomic sequencing data are not publicly available. Researchers wishing to access the raw sequencing data for academic and scientific purposes must first obtain appropriate IRB approval at their respective institutions. After receiving IRB authorization, requests for data access may be directed to the corresponding author, who will facilitate data sharing in accordance with the relevant ethical guidelines.

## Supplementary Materials

The following supporting information can be downloaded at https://www.mdpi.com/article/10.3390/microorganisms14102211/s1: Supplementary Methods, Figure S1: Relative Abundance of Various Genera and Species at Different Levels of Kynurenine Metabolite from the Kynurenine Pathway. Figure S2: Relative Abundance of Various Genera and Species at Different Levels of Kynurenic Acid Metabolite from the Kynurenine Pathway. Figure S3: Relative Abundance of Various Genera and Species at Different Levels of Nicotinuric Acid Metabolite from the Kynurenine Pathway. Figure S4: Relative Abundance of Various Genera and Species at Different Levels of Trigonelline Metabolite from the Kynurenine Pathway. Figure S5: LEfSe (Linear Discriminant Analysis Effect Size) Analysis and Cladogram Construction for the Microbiome Associated with Kynurenine. Figure S6: The Comparison of Standardized Species Relative Abundance Between High and Low Levels of Kynurenine. Figure S7: LEfSe (Linear Discriminant Analysis Effect Size) Analysis of Gut Metabolic Modules Based on the Level of Kynurenine. Figure S8: LEfSe (Linear Discriminant Analysis Effect Size) Analysis and Cladogram Construction for the Microbiome Associated with Kynurenic Acid. Figure S9: The Comparison of Standardized Species Relative Abundance Between High and Low Levels of Kynurenic Acid. Figure S10: LEfSe (Linear Discriminant Analysis Effect Size) Analysis of Gut Metabolic Modules Based on the Level of Kynurenic Acid. Figure S11: LEfSe (Linear Discriminant Analysis Effect Size) Analysis and Cladogram Construction for the Microbiome Associated with Nicotinuric Acid. Figure S12: The Comparison of Standardized Species Relative Abundance Between High and Low Levels of Nicotinuric Acid. Figure S13: LEfSe (Linear Discriminant Analysis Effect Size) Analysis of Gut Metabolic Modules Based on the Level of Nicotinuric Acid. Figure S14: LEfSe (Linear Discriminant Analysis Effect Size) Analysis and Cladogram Construction for the Microbiome Associated with Trigonelline. Figure S15: The Comparison of Standardized Species Relative Abundance Between High and Low Levels of Trigonelline. Figure S16: LEfSe (Linear Discriminant Analysis Effect Size) Analysis of Gut Metabolic Modules Based on the Level of Trigonelline.
