# Supplementary material for: Identification of Gut Microbiome Signatures Associated with the Kynurenine Pathway in Tryptophan Metabolism in Patients Undergoing Hemodialysis
Source: Microorganisms. 2026 Oct 1;14(10):2211. doi: 10.3390/microorganisms14102211 (PMC13707831; doi:10.3390/microorganisms14102211)

Supplementary Material

Identification of gut microbiome signatures associated with kynurenine pathway in tryptophan metabolism in patients undergoing hemodialysis

Supplementary Methods

S1. Study population and data collection (expanded)

S1.1 Study design and participants

This study enrolled 85 stable hemodialysis (HD) patients from the dialysis center of Kaohsiung Medical University Hospital, between August 2017 and February 2018. Patients with active malignancies or antibiotic use within three months prior to enrollment were excluded. All participants received maintenance HD using high-flux dialyzers for 3.5–4 hours per session, three times per week.

The study protocols were approved by the Institutional Review Board of Kaohsiung Medical University (KMUHIRB-E(I)-20160095 and KMUHIRB-E(I)-20180118). Written informed consent was obtained from all participants prior to study enrollment.

S1.2 Clinical variables and comorbidity definitions

Demographic characteristics, medication use (including proton pump inhibitors), medical history, and laboratory data were extracted from hospital records. Diabetes mellitus was defined as HbA1c ≥6.5% or current use of antidiabetic medications. Hypertension was defined as systolic/diastolic blood pressure ≥140/90 mmHg or use of antihypertensive treatment. Coronary artery disease was defined by documented angina, ischemic electrocardiographic changes, previous myocardial infarction, or history of coronary artery bypass surgery or angioplasty.

Blood samples were collected after an overnight fast and prior to scheduled HD sessions. Serum albumin and high-sensitivity C-reactive protein (hs-CRP) values were obtained from laboratory records within 30 days preceding the HD procedure and analyzed in the hospital central laboratory.

S2. Targeted LC–MS/MS measurement of kynurenine-pathway metabolites

S2.1 Sample processing and metabolite extraction

For metabolite extraction, 150 μL of methanol containing internal standard (50 ng/L) was added to 30 μL of serum. The mixture was incubated at 4°C for 30 minutes and centrifuged at 5,000 rpm at 4°C for 5 minutes. The supernatant was collected and mixed with 120 μL of

water containing internal standard. All samples were filtered through 0.2 μm PVDF membranes prior to instrumental analysis.

S2.2 Calibration standards and quality control

Analytical standards were prepared using dilution series derived from a primary mixture containing eight target compounds, including kynurenine, kynurenic acid, nicotinuric acid, and trigonelline (10 mg/L each). Final calibration concentrations ranged from 2.5 to 5,000 ng/mL in 50% methanol. Quality control samples were prepared at 200 ng/mL for all analytes and processed using the same procedures as calibration standards.

S2.3 Chromatographic and mass spectrometric conditions

Targeted metabolite analysis was performed using a SHIMADZU LC-20ADxr system coupled to a SCIEX QTrap® 5500 mass spectrometer. Separation was achieved on an HSS-T3 column (2.1 × 100 mm, 1.7 μm). The mobile phases consisted of 0.1% formic acid in water (A) and 0.1% formic acid in acetonitrile (B), delivered at a flow rate of 0.35 mL/min.

The gradient program started at 15% B (0–0.3 min), increased to 55% B (0.3–9.5 min), and reached 99% B (9.5–10.5 min). The sample tray and column temperatures were maintained at 4°C and 40°C, respectively. Analyses were performed in dual positive/negative electrospray ionization modes with an injection volume of 1 μL.

S3. Fecal sample collection and DNA extraction

S3.1 Fecal sample collection and transport

Fecal samples were collected from each participant and immediately frozen. Samples were transported to the laboratory (Germark Biotechnology, Taichung, Taiwan) in cooler bags within 24 hours of collection.

S3.2 DNA extraction and quality assessment

Bacterial DNA was extracted using the QIAamp Fast DNA Stool Mini Kit (Qiagen, MD, USA). DNA concentration and purity were assessed using NanoDrop ND-1000 spectrophotometry and agarose gel electrophoresis. Extracted DNA samples were stored at −80°C prior to library preparation.

S4. Shotgun metagenomics sequencing and bioinformatics pipeline

S4.1 Library preparation and sequencing

Approximately 500 ng of extracted DNA was fragmented to ~350 bp using the Covaris S2 system. Sequencing libraries were constructed using the Illumina DNA Prep Kit. Paired-end sequencing (150 bp) was performed on the Illumina NovaSeq 6000 platform. Sequencing depth achieved an average throughput of \~8–9 Gb per sample (ranging from 40 to 85 million raw reads per sample).

S4.2 Raw read quality control and host contamination removal

Raw sequencing reads were quality controlled using the Kneaddata workflow. Trimmomatic was used to remove adapter sequences, trim low-quality bases, and discard short reads. Host-derived sequences were removed by aligning reads to the human reference genome (hg38) and PhiX genome using Bowtie2. Low-complexity reads were filtered using Komplexity with default parameters.

S4.3 Metagenome assembly, taxonomic profiling, and functional annotation

Quality-controlled reads were assembled into contigs using MEGAHIT. Contig-based taxonomic classification was performed using the MMSeqs2 easy-taxonomy pipeline. Contig abundance was estimated based on contig depth calculated using the jgi_summarize_bam_contig_depths script following Bowtie2 alignment of reads to contigs.

Open reading frames were predicted and annotated using Prokka, including assignment of Clusters of Orthologous Groups and Enzyme Commission numbers. ORFs were further annotated with KEGG Orthology identifiers, Carbohydrate Active enZyme families, antibiotic resistance genes, and virulence factors using the MMSeqs2 easy-search pipeline. Pathway reconstruction was conducted using MinPath based on KEGG and MetaCyc pathway annotations.

S4.4 Metagenome binning and MAG assessment

Metagenomic binning was performed using MetaBAT to generate metagenome-assembled genomes (MAGs). MAG quality and taxonomy were assessed using the lineage-specific workflow of CheckM. Taxonomic assignments were further refined by voting at the contig level within each bin.

S5. Microbiome preprocessing and derived features

S5.1 Data preprocessing

Microbiome data were imported and processed using the R package phyloseq. Processing steps included data import, pruning, transformation to relative abundance, aggregation, and

grouping. Taxa with summed relative abundance $<1\times10^{-5}$ or missing taxonomic annotations were removed. Remaining taxa were aggregated at the species level.

S5.2 Definition of microbial and functional features

Species-level metagenomic species (MGS) profiles were used for microbial analyses. Functional pathway profiles were derived from genomic metabolic modules (GMM) reconstructed from annotated metagenomic data. For each metabolite, participants were dichotomized into high and low groups based on the median metabolite concentration.

S6. Statistical analysis details

S6.1 Baseline comparisons

Baseline demographic and clinical characteristics were compared between high and low metabolite groups. Continuous variables were analyzed using Student's t-test or Wilcoxon rank-sum test, and categorical variables were analyzed using chi-square tests. These analyses were conducted using the compareGroups function in R.

S6.2 Diversity analyses

Alpha diversity indices (Shannon, Simpson, inverse Simpson) were calculated from species-level relative abundance profiles. Beta diversity was assessed using Bray–Curtis dissimilarity and visualized by principal coordinate analysis (PCoA) using the ade4 package. Differences in microbial community composition between groups were evaluated using PERMANOVA via the adonis2 function in the vegan package.

S6.3 Association analyses and feature selection

Associations between serum metabolite concentrations (kynurenine, kynurenic acid, nicotinuric acid, and trigonelline) and gut microbial features were evaluated using partial Spearman rank correlation analyses adjusted for age and sex across all 85 participants. Correlations were computed separately across 867 retained bacterial species and 100 genomic metabolic modules (GMMs). To control for multiple testing, p-values were adjusted within each metabolite-by-data-layer family using the Benjamini-Hochberg false discovery rate (FDR) procedure. Associations with $q<0.05$ were defined as FDR-significant, whereas associations with $0.05 \leq q <0.20$ were reported strictly as exploratory findings. Exploratory feature rankings were additionally evaluated using linear discriminant analysis effect size (LEfSe) with an LDA score threshold of 2.0; because default LEfSe p-values do not control

the false discovery rate, LEfSe outputs are reported with nominal p-values and LDA scores and are not classified as FDR-significant.

To estimate the collective descriptive variance in metabolite concentrations accounted for by selected microbial taxa, participants were first dichotomized into high and low groups based on median metabolite concentrations. Candidate taxa were screened using LEfSe with log10 p-value transformation, Wilcoxon and Kruskal–Wallis significance cutoffs of 0.05, multigroup stratification, and an LDA score cutoff of 2.1. Taxa meeting these criteria were further filtered by requiring a nominal equal-variance two-sample comparison p < 0.05 at the species level. The retained species for each metabolite were then entered jointly into full-sample Ordinary Least Squares (OLS) linear regression models across all 85 participants, with untransformed serum metabolite concentrations as the dependent variable and log1p-transformed species relative abundances as independent variables. The conventional coefficient of determination ($R^2 = 1 - SSE/SST$) was calculated for each model. Because both feature selection and coefficient estimation were performed within the same dataset, these R^2 values represent descriptive in-sample goodness-of-fit and should not be interpreted as out-of-sample predictive performance or causal variance attributable to the microbiome.

To assess the stability of observed species and functional module associations against broader clinical confounding, sensitivity analyses were conducted using expanded partial Spearman correlation models. In addition to age and sex, models adjusted for five key clinical and laboratory covariates: diabetes mellitus, hypertension, coronary artery disease, proton pump inhibitor (PPI) use, and serum albumin (complete data across all N = 85 participants). Benjamini-Hochberg FDR correction was re-applied separately within each metabolite and data-layer family.

S6.4 Differential abundance and pathway analyses

Linear discriminant analysis effect size (LEfSe) was applied to identify taxa differentially abundant between high and low metabolite groups and to generate cladograms illustrating phylogenetic relationships. LEfSe analysis was also applied to GMM pathway profiles to identify differentially enriched metabolic functions.

S6.5 Software

All statistical analyses were conducted using R software. Microbiome data visualization was performed using ggplot2. Bioinformatics analyses were conducted by Germark Biotechnology.

Supplementary Figures

Figure S1. Relative Abundance of Various Genera and Species at Different Levels of Kynurenine Metabolite from the Kynurenine Pathway.

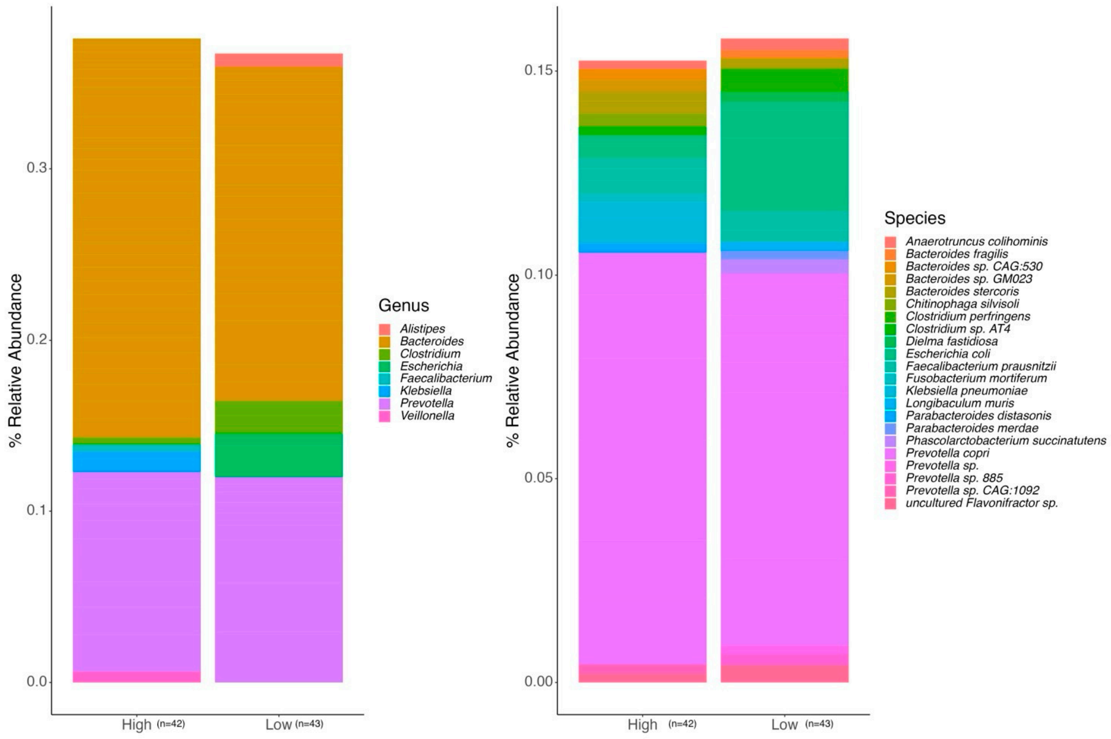

Figure S2. Relative Abundance of Various Genera and Species at Different Levels of Kynurenic Acid Metabolite from the Kynurenine Pathway.

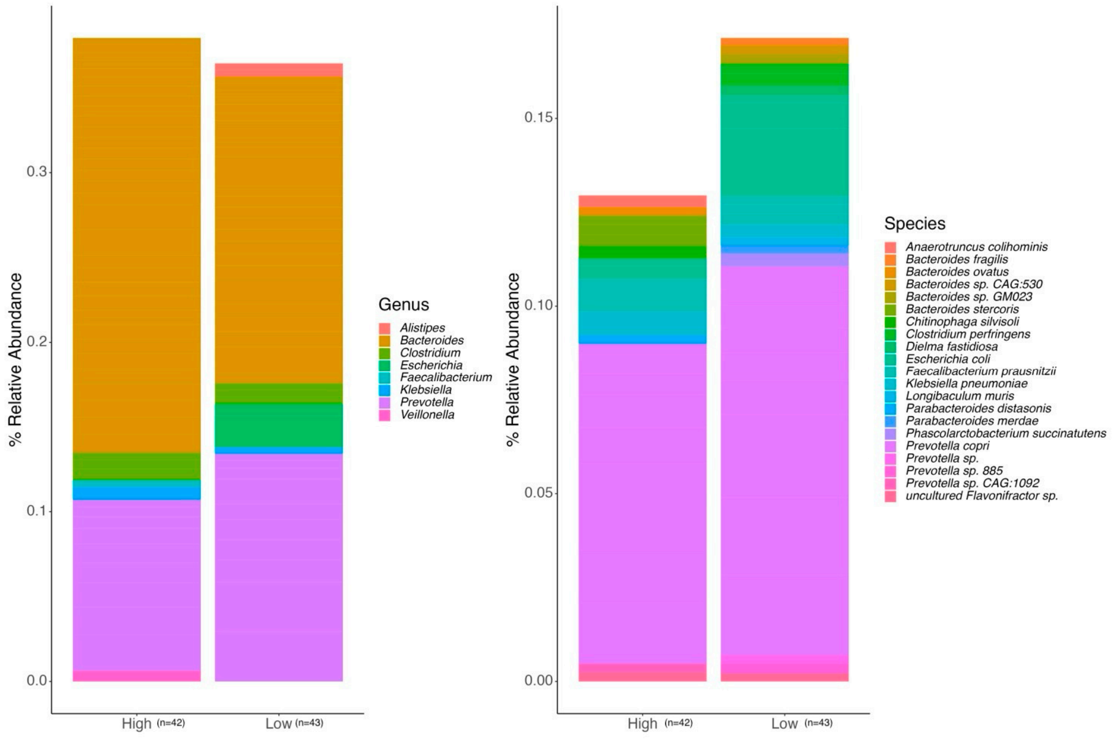

Figure S3. Relative Abundance of Various Genera and Species at Different Levels of Nicotinuric Acid Metabolite from the Kynurenine Pathway.

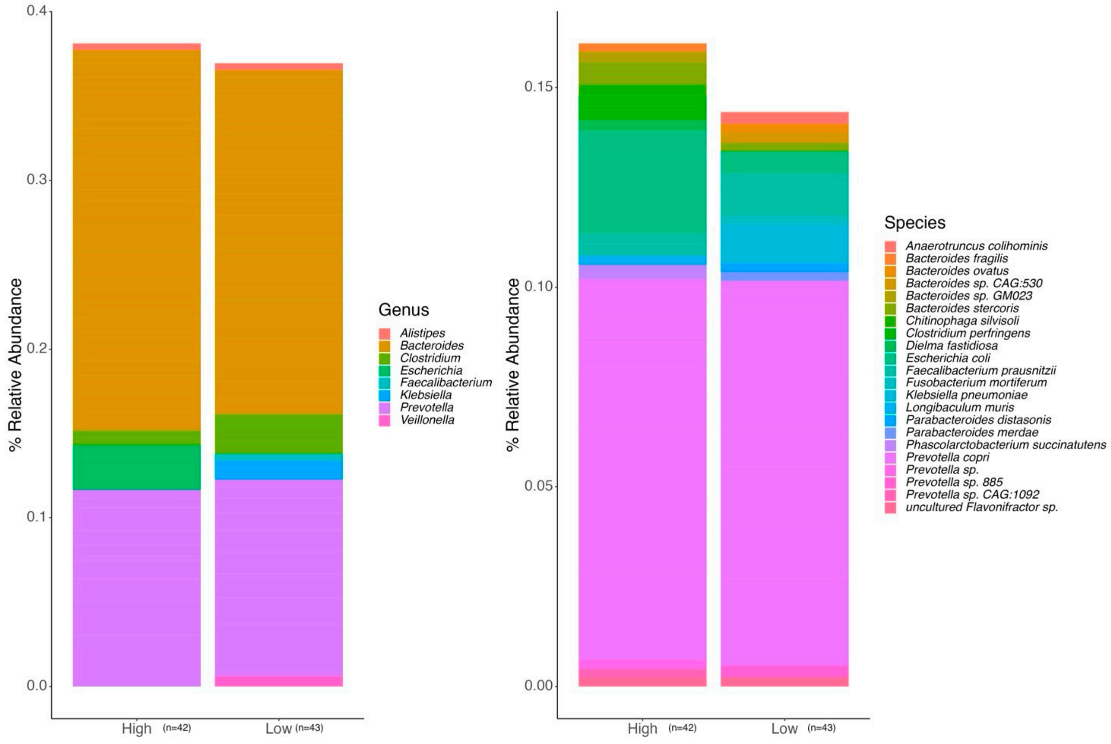

Figure S4. Relative Abundance of Various Genera and Species at Different Levels of Trigonelline Metabolite from the Kynurenine Pathway.

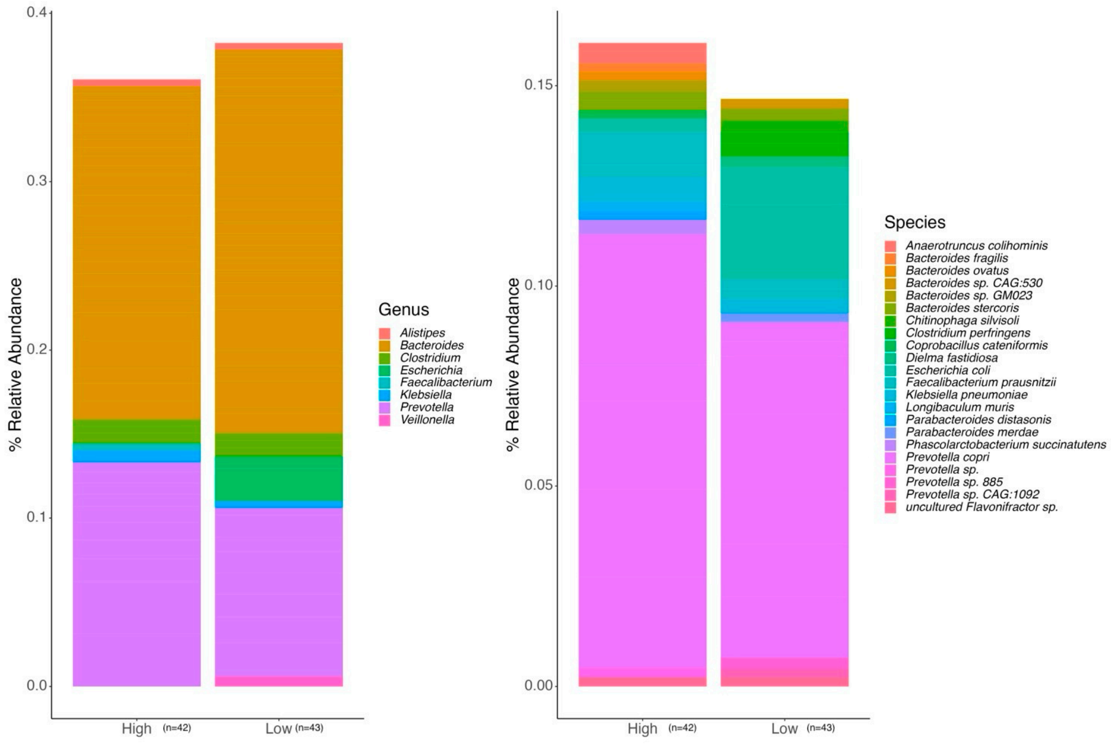

Figure S5. LEfSe (Linear Discriminant Analysis Effect Size) Analysis and Cladogram Construction for the Microbiome Associated with Kynurenine.

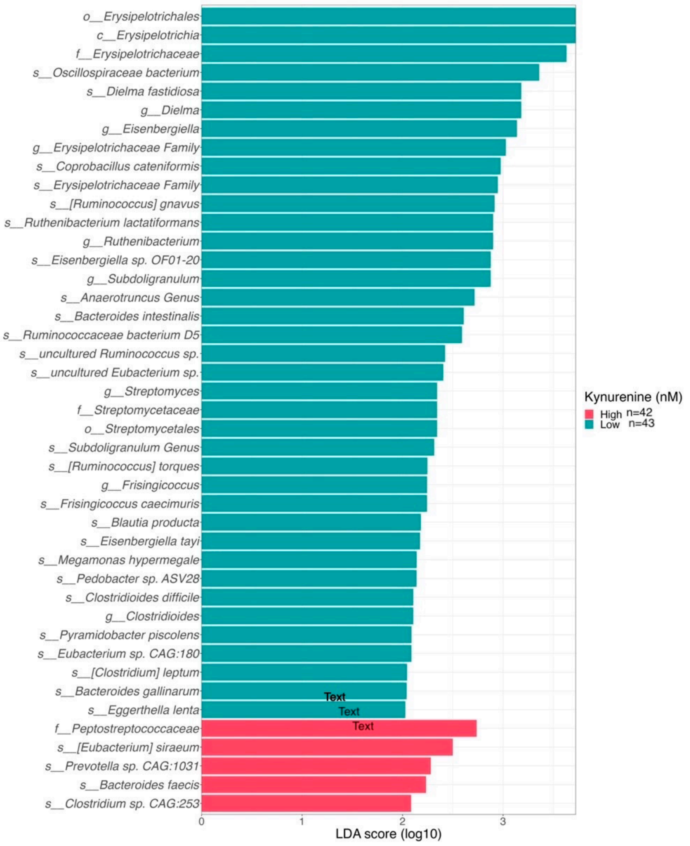

(A)

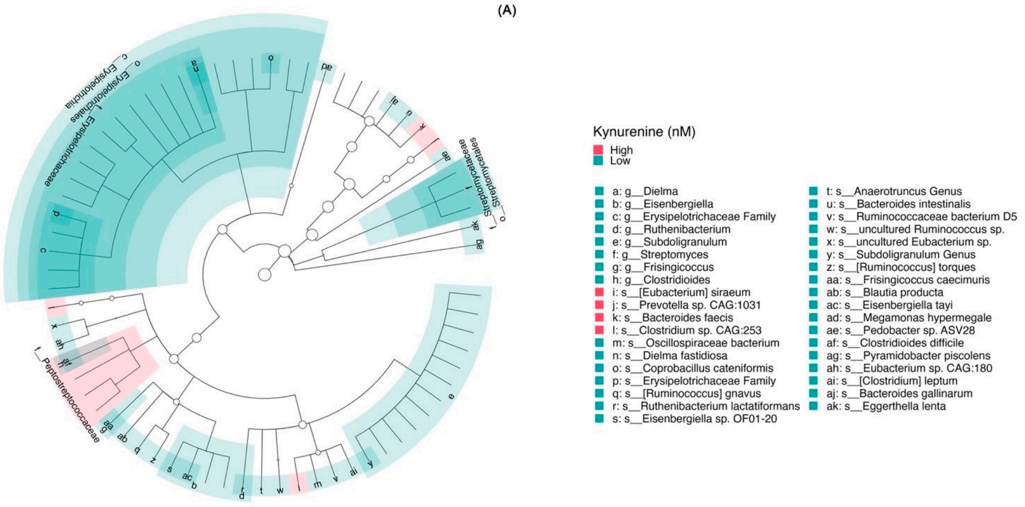

(B)

Figure S6. The Comparison of Standardized Species Relative Abundance Between High and Low Levels of Kynurenine.

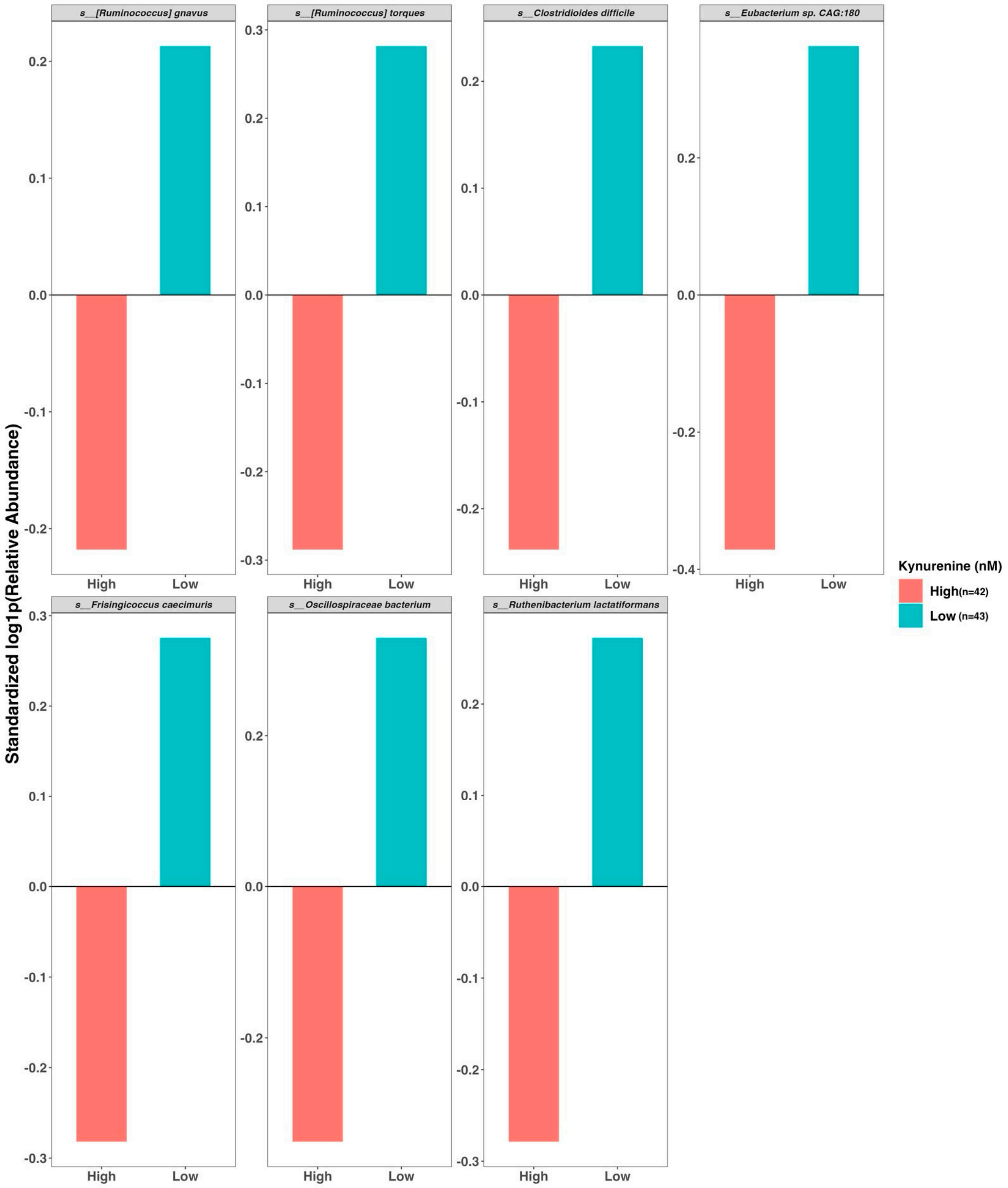

Figure S7. LEfSe (Linear Discriminant Analysis Effect Size) Analysis of **Gut Metabolic Modules Based on the Level of Kynurenine.**

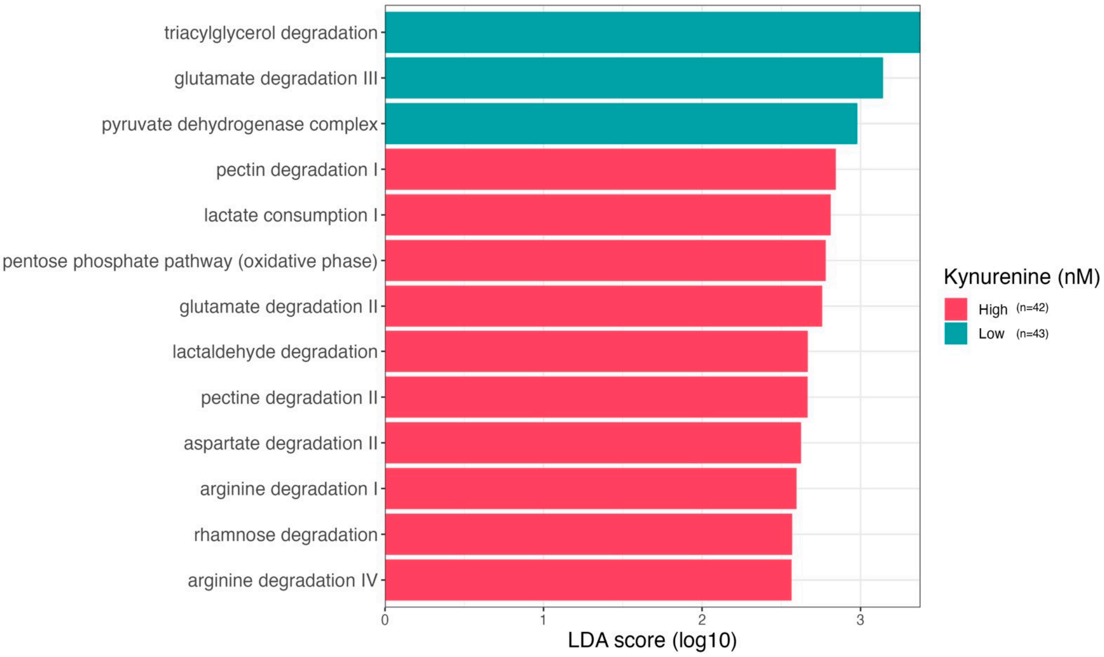

Figure S8. LEfSe (Linear Discriminant Analysis Effect Size) Analysis and Cladogram Construction for the Microbiome Associated with Kynurenic Acid.

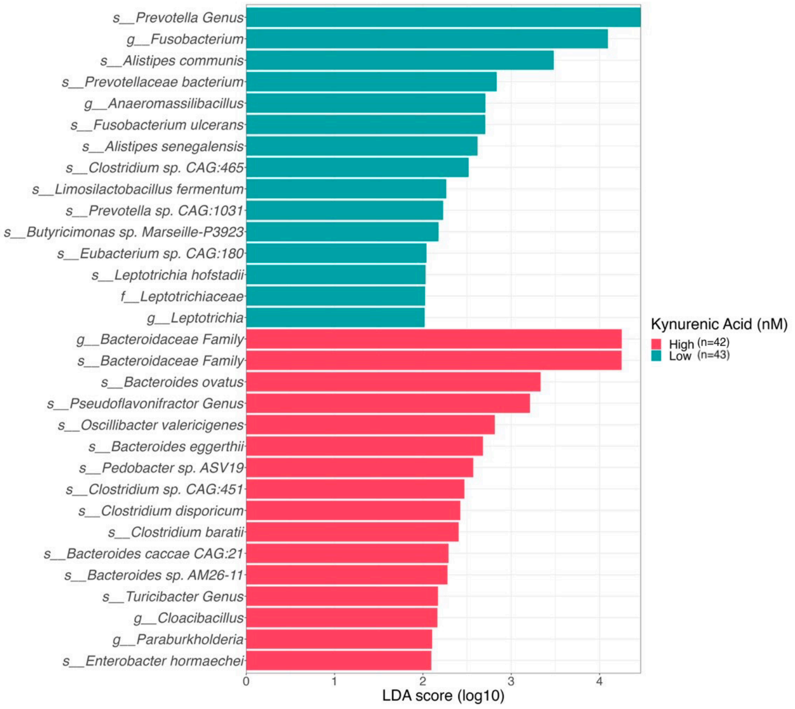

(A)

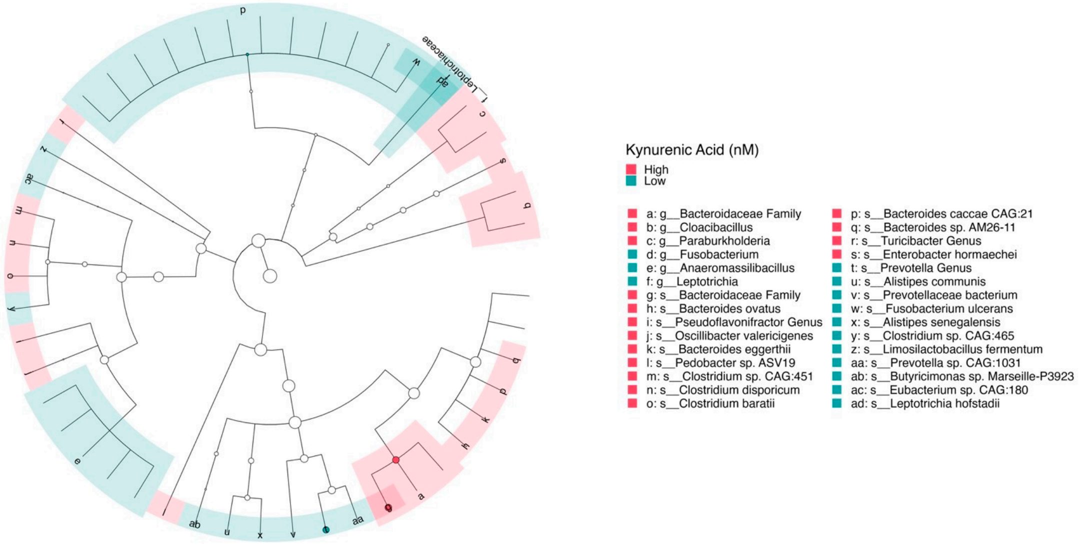

(B)

Figure S9. The Comparison of Standardized Species Relative Abundance Between High and Low Levels of Kynurenic Acid.

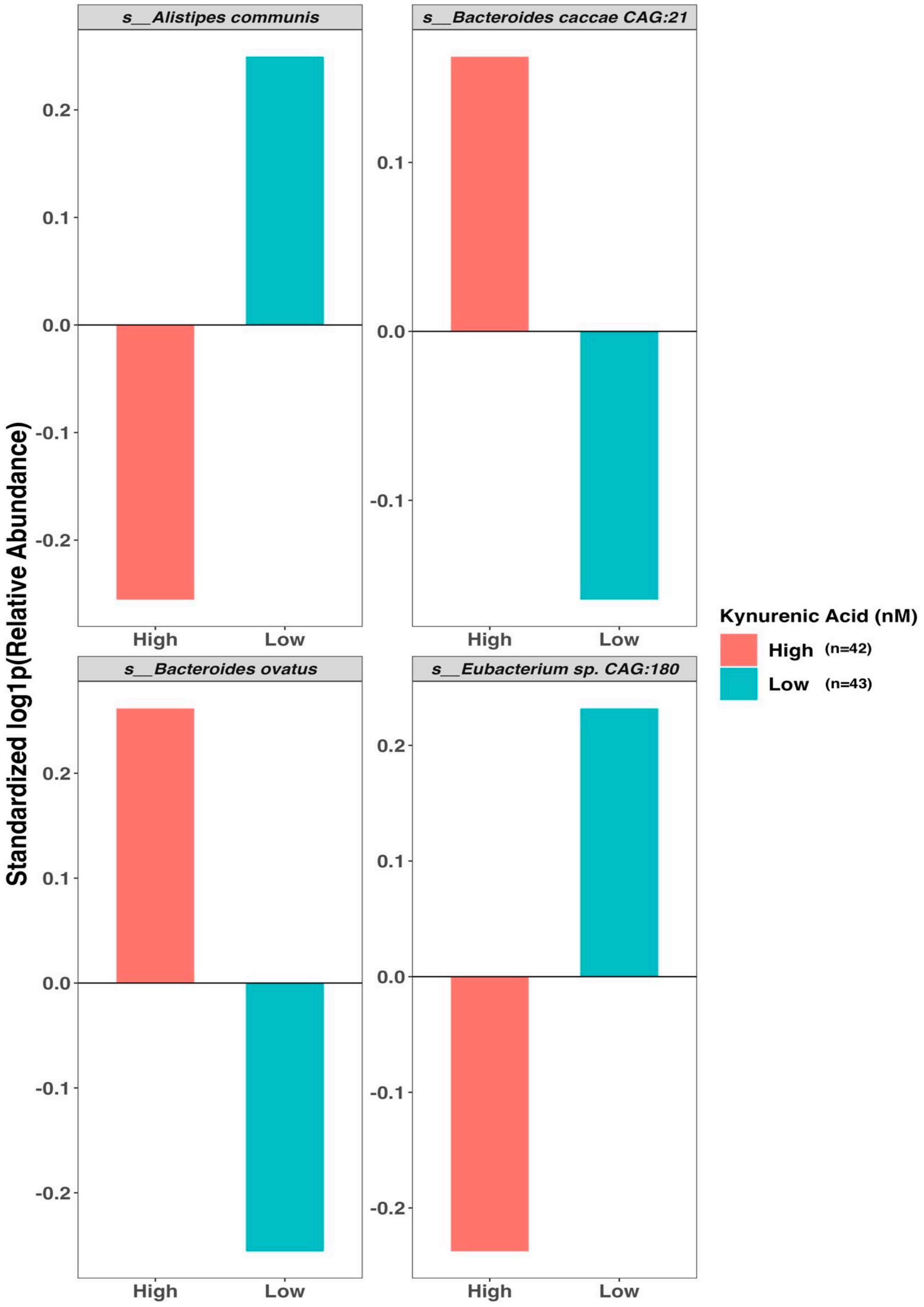

Figure S10. LEfSe (Linear Discriminant Analysis Effect Size) Analysis of Gut Metabolic Modules Based on the Level of Kynurenic Acid.

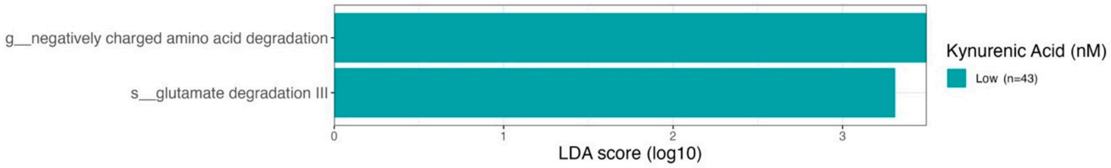

Figure S11. LEfSe (Linear Discriminant Analysis Effect Size) Analysis and Cladogram Construction for the Microbiome Associated with Nicotinuric Acid.

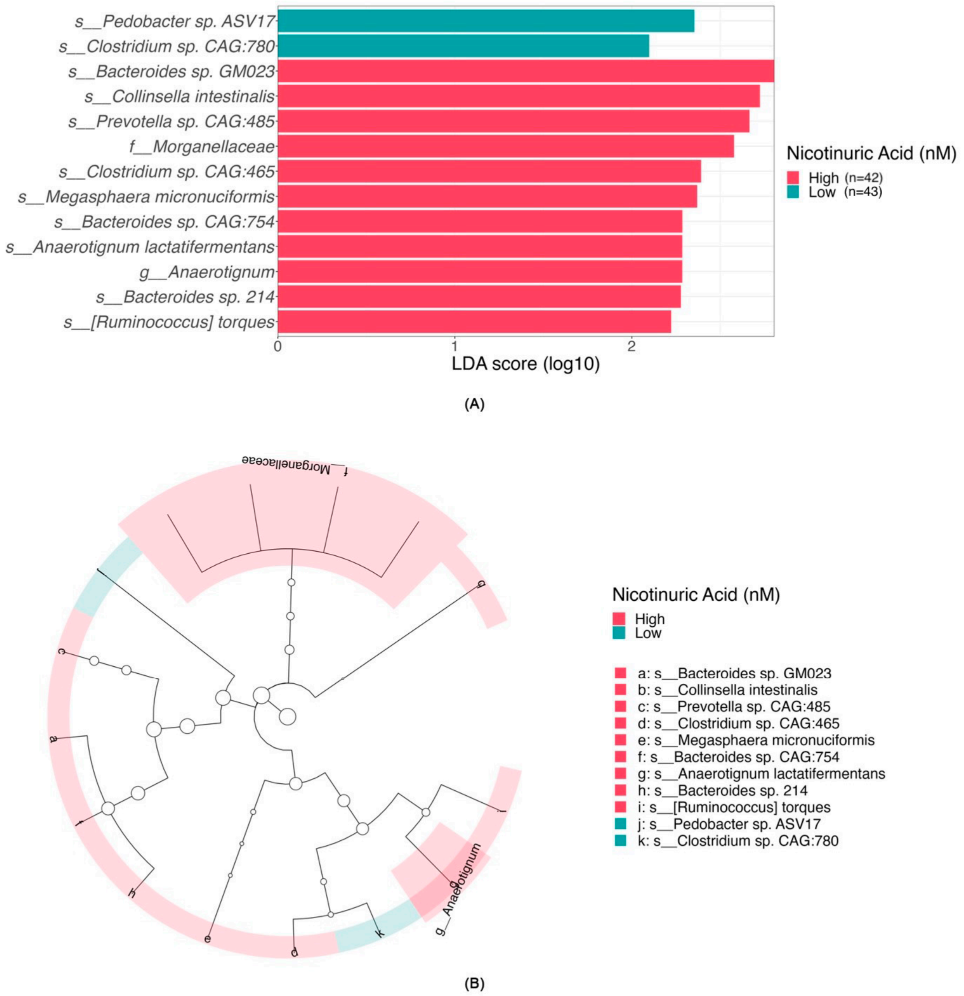

Figure S12. The Comparison of Standardized Species Relative Abundance Between High and Low Levels of Nicotinuric Acid.

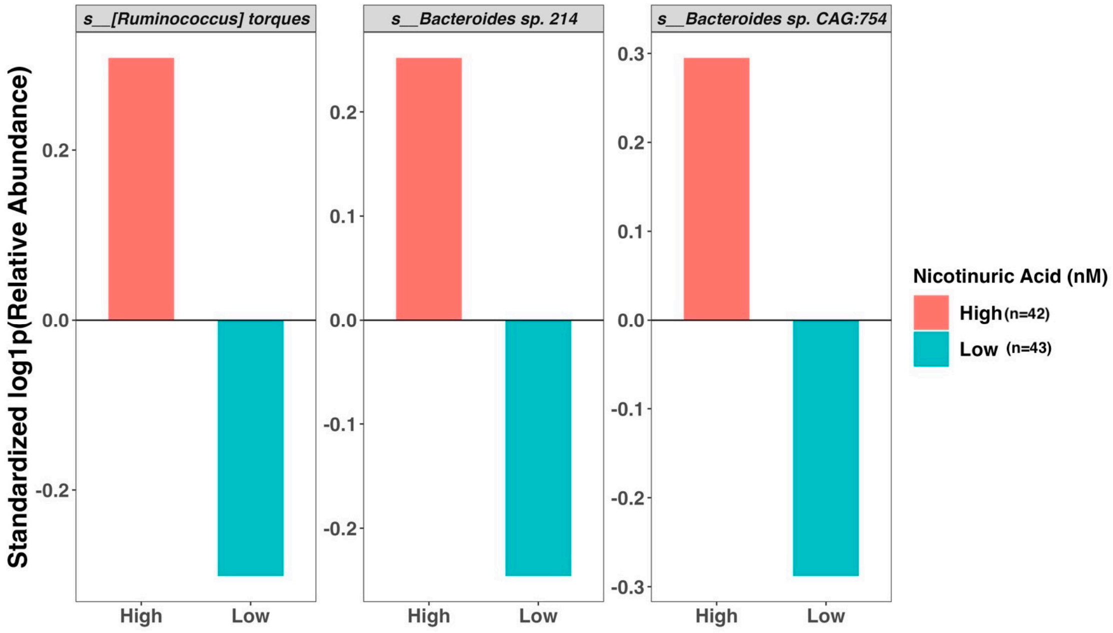

Figure S13. LEfSe (Linear Discriminant Analysis Effect Size) Analysis of Gut Metabolic Modules Based on the Level of Nicotinuric Acid.

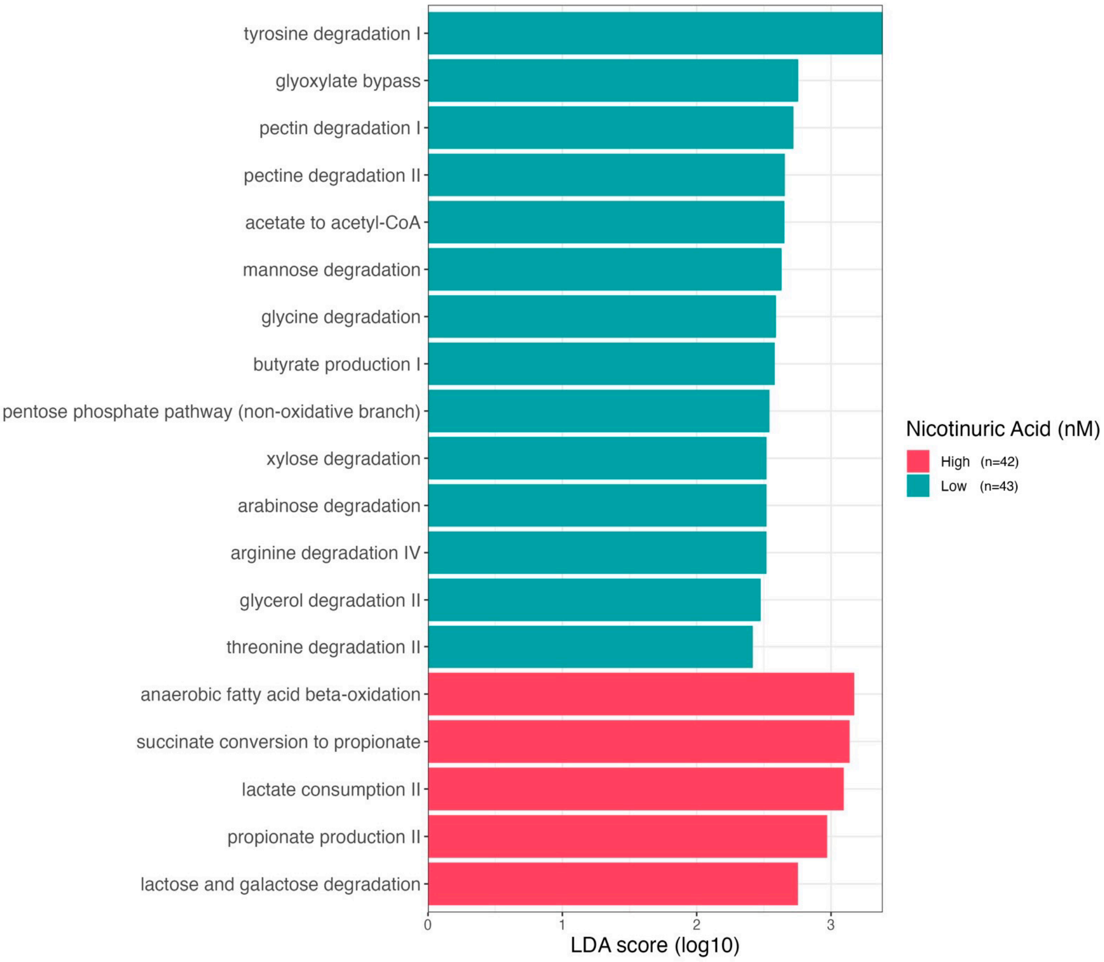

Figure S14. LEfSe (Linear Discriminant Analysis Effect Size) Analysis and Cladogram Construction for the Microbiome Associated with Trigonelline.

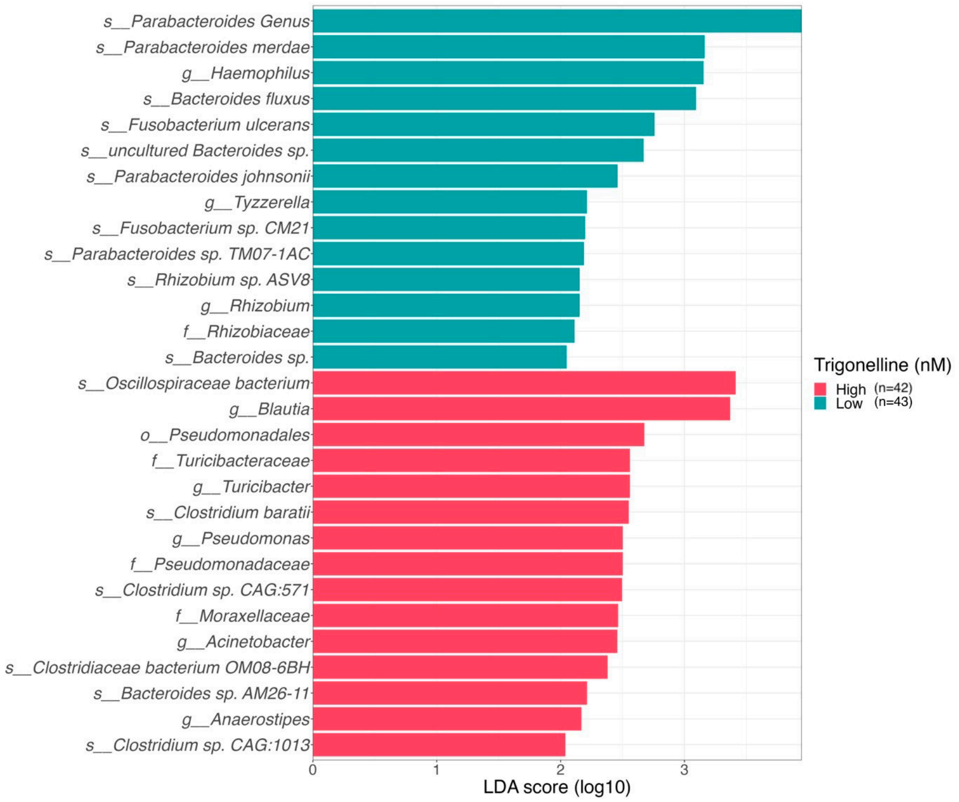

(A)

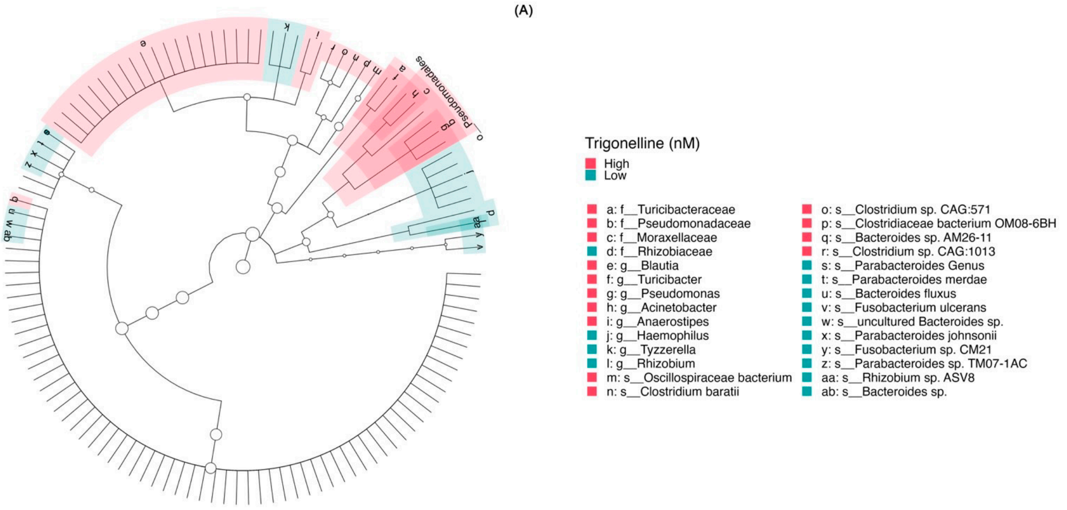

(B)

Figure S15. The Comparison of Standardized Species Relative Abundance Between High and Low Levels of Trigonelline.

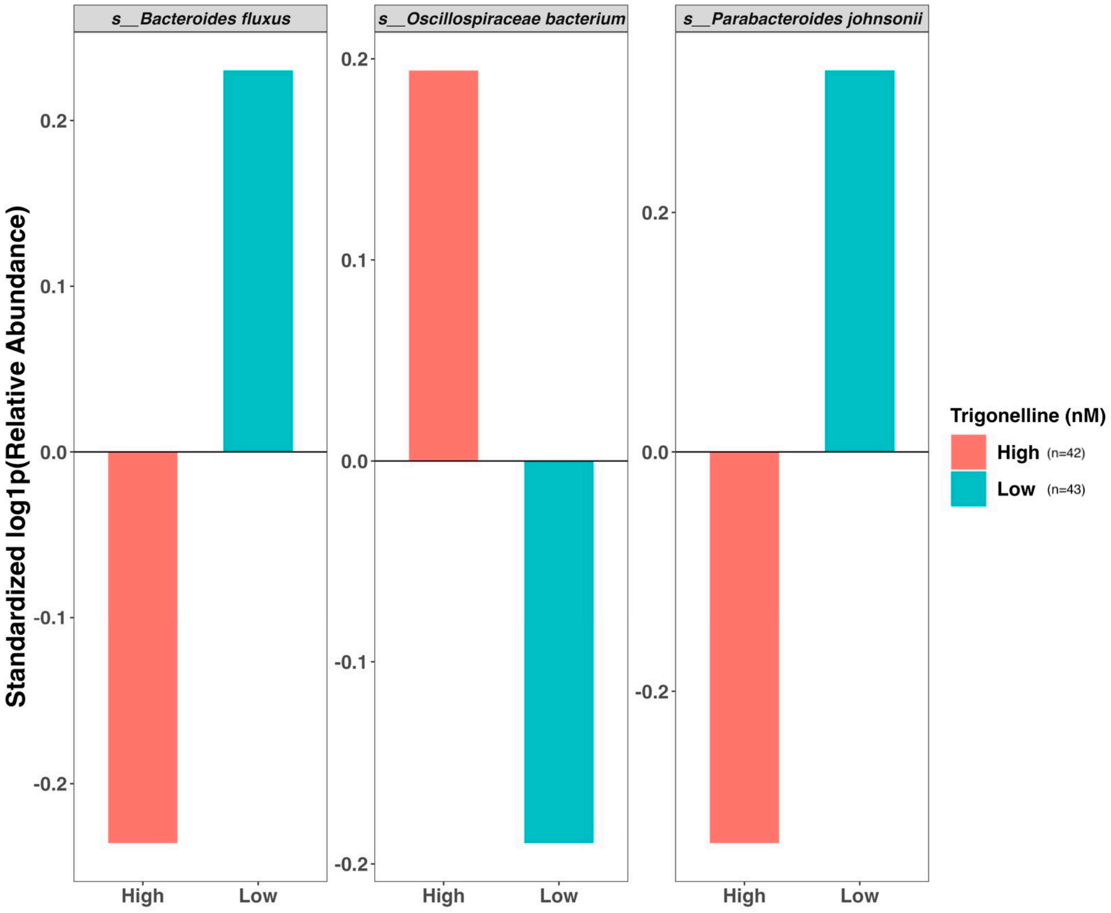

Figure S16. LEfSe (Linear Discriminant Analysis Effect Size) Analysis of Gut Metabolic Modules Based on the Level of Trigonelline.

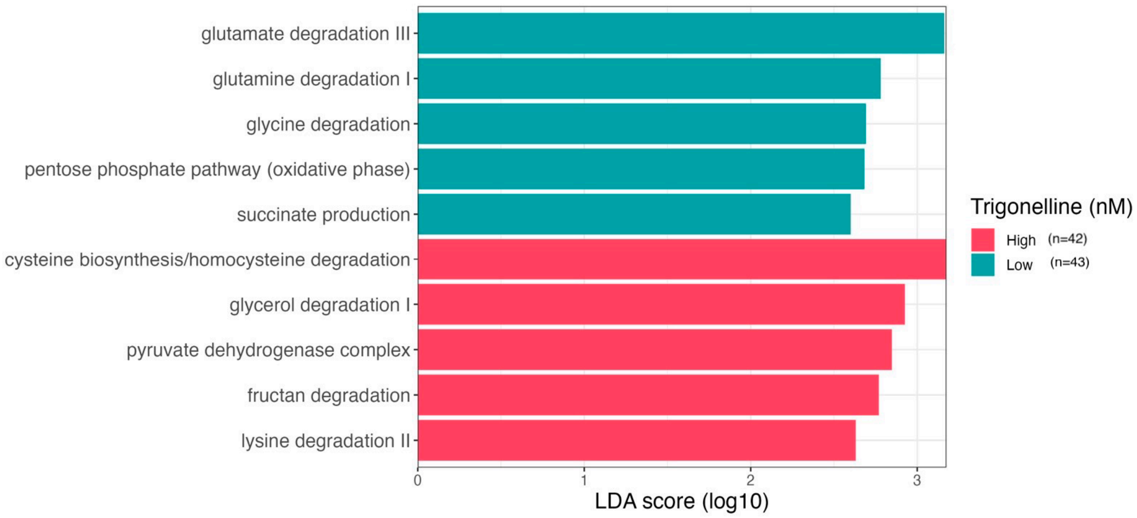

Supplement: Supplementary file 1 [file microorganisms-14-02211-s001.zip › microorganisms-4575496-supplementary.pdf]
